# Des-Aspartate-Angiotensin I Attenuates Mortality of Mice Exposed to Gamma Radiation via a Novel Mechanism of Action

**DOI:** 10.1371/journal.pone.0138009

**Published:** 2015-09-17

**Authors:** Hong Wang, Gautam Sethi, Weng-Keong Loke, Meng-Kwoon Sim

**Affiliations:** 1 Department of Pharmacology, Yong Loo Lin School of Medicine, National University of Singapore, Block MD11, 10 Medical Drive, Singapore, Singapore; 2 Agent Diagnostic and Therapeutics Laboratory, Defence & Environmental Research Institute, DSO National Laboratories, 11 Stockport Road, Singapore, Singapore; ENEA, ITALY

## Abstract

ACE inhibitors and ARBs (angiotensin receptor blockers) have been shown to attenuate radiation injuries in animal models of lethal gamma irradiation. These two classes of drug act by curtailing the actions of angiotensin II-linked inflammatory pathways that are up-regulated during gamma radiation in organ systems such as the brain, lung, kidney, and bone marrow. ACE inhibitors inhibit ACE and attenuate the formation of angiotensin II from angiotensin I; ARBs block the angiotensin AT_1_ receptor and attenuate the actions of angiotensin II that are elicited through the receptor. DAA-I (des-aspartate-angiotensin I), an orally active angiotensin peptide, also attenuates the deleterious actions of angiotensin II. It acts as an agonist on the angiotensin AT_1_ receptor and elicits responses that oppose those of angiotensn II. Thus, DAA-I was investigated for its anticipated radioprotection in gamma irradiated mice. DAA-I administered orally at 800 nmole/kg/day for 30 days post exposure (6.4 Gy) attenuated the death of mice during the 30-day period. The attenuation was blocked by losartan (50 nmole/kg/day, i.p.) that was administered sequential to DAA-I administration. This shows that the radioprotection was mediated via the angiotensin AT_1_ receptor. Furthermore, the radioprotection correlated to an increase in circulating PGE_2_ of surviving animals, and this suggests that PGE_2_ is involved in the radioprotection in DAA-I-treated mice. At the hematopoietic level, DAA-I significantly improved two syndromes of myelosuppression (leucopenia and lymphocytopenia), and mice pre-treated with DAA-I prior to gamma irradiation showed significant improvement in the four myelodysplastic syndromes that were investigated, namely leucopenia, lymphocytopenia, monocytopenia and thrombocytopenia. Based on the known ability of PGE_2_ to attenuate the loss of functional hematopoietic stem and progenitor cells in radiation injury, we hypothesize that PGE_2_ mediated the action of DAA-I. DAA-I completely attenuated the increase in circulating level of two inflammatory cytokines, TNFα and IL-6, in irradiated mice; and this shows that DAA-I exerted additional anti-inflammatory actions, which could also have contributed to its radioprotection. These findings show that DAA-I acts via a novel mechanism of action on the angiotensin AT_1_ receptor to specifically release PGE_2_, which mediates radioprotection in the gamma irradiated mice.

## Introduction

Gamma radiation induces radiolysis of tissue water and generates reactive oxygen species (ROS). This results in inflammatory responses that are characterized by the production of pro-inflammatory cytokines in mice that had been exposed to gamma radiation. The mouse model has been used to study the radioprotective effects of biological compounds with known antioxidant properties, and significant protection in terms of attenuation of cellular inflammatory processes and death have been reported [1–5]. The present study describes the radioprotective action of an endogenous angiotensin peptide, des-aspartate-angiotensin I (DAA-I). Early studies show that DAA-I is efficacious in animal model of inflammatory diseases in which angiotensin II has been implicated [6–13]. Angiotensin II is also implicated in radiation-induced organ damage as ACE inhibitors (which curtail angiotensin II formation from angiotensin I) and ARBs (which block angiotensin II actions at the angiotensin AT_1_ receptor) have been shown to ameliorate the radiation-induced damage in the kidney [14], lung [15] and brain [16] of rats. It is, thus, anticipated that DAA-I would be an effective radioprotector that acts to attenuate an underlying cause of radiation-induced pathologies and death. DAA-I acts as an agonist on the angiotensin AT_1_ receptor and mediates responses opposing the deleterious effects of angiotensin II. It is a prototype of an angiotensin AT_1_ receptor agonist [17]. In this respect, losartan, an angiotensin AT_1_ receptor blocker, was investigated with regard to its effect on the anticipated radioprotective action of DAA-I. The actions of DAA-I are indomethacin sensitive and this indicates that prostaglandins mediate its actions (6–8, 11, 13). Based on the finding that PGE_2_ promotes intestinal crypt stem cell survival and proliferation after radiation injury(18), the possibility of PGE_2_ acting as a mediator of DAA-I actions was also investigated by measuring the level of circulating PGE_2_ in the animals used in the experiments. The present study is a first investigation on an angiotensin peptide with an established pharmacology and mechanism of action.

## Materials and Methods

### Reagents

Des-aspartate-angiotensin I (DAA-I) was purchased from Bachem AG, Switzerland. Losartan potassium was purchased from Sigma-Aldrich, USA. DAA-I was prepared as a stock solution of 1600 nmole/mL (in sterile water) and stored frozen till used. It was diluted 10 times prior to use. Similarly losartan potassium was prepared as a stock solution of 100 nmole/mL (in sterile saline) and stored frozen till used. It was diluted 10 times prior to use. Prostaglandin E (PGE) metabolite EIA kits were purchased from Cayman Chemical Company, USA, and DuoSet ELISA kits for mouse IL-6 (DY406) and TNF-a (DY410) from R&D system, USA.

### Animals

7–8 week Balb/c female mice were obtained from In Vivos, Singapore, and housed in Comparative Medicine Facility, National University of Singapore, with free access to water and feed.

### Ethics Statement

All animal procedures were carried out in accordance with the protocols and guidelines of the Institutional Animal Use and Care Committee of the National University of Singapore. The protocol was approved by the Institutional Animal Use and Care Committee of the National University of Singapore (Permit Number: IACUC 081/12).

### Total body gamma-irradiation of mice

Forty mice were randomly divided into 4 groups of 10 mice. Animals were administered daily vehicle or drug/s on day 1 for a period of 30 days. Mice in the first group served as control (Control-1). The mice in this group were orally administered (by gavage) 0.1 ml vehicle (water) and were not exposed to gamma irradiation. The mice in the other three groups were subjected to 6.4 Gy of total body gamma irradiation (TBI). The first group of mice that were exposed to TBI were orally administered 0.1 ml DAA-I solution equivalent to 800 nmole/kg of body weight; mice in the second group were similarly administered the same dose of DAA-I followed with intraperitoneal injection of 0.1 ml losartan solution equivalent to 50 nmole/kg body weight; and mice in the third group were orally administered 0.1 ml of vehicle and served as a second control (Control-2).The scheme of the animal protocol is given in Table 1.

**Table 1:** Drug administration protocol of non-irradiated and irradiated mice.

Group	TBI (6.4 Gy)	Vehicle (0.1 ml)	DAA-I (0.1 ml)	Losartan (0.1 ml)
Control- 1	-	+	-	-
Group 1	+	-	+	-
Group 2	+	-	+	+
Control- 2	+	+	-	-

An additional group of 6 mice were pre-treated with the same dose of DAA-I for 14 days before TBI, and treatment with DAA-I was continued for a further 15 days post TBI. The dose of DAA-I was selected based on early studies showing that 800 nmole/kg DAA-I administered orally was effective in combating cardiac hypertrophy [13] and glomerulosclerosis and renal failure [9]. The dose of losartan was selected based on an early study showing that at 50 nmole/kg, losartan had no pharmacological action per se but was able to attenuate the action of DAA-I [7]. A humane endpoint was used in the present study. The weight and general health of each animal was monitored daily. The house veterinarian was consulted regarding the survival status of an animal when it lost more than 10% of its weight and looked sick. Sick animals that had either one or a combination of the following symptoms: nose bleeding, weakness and in moribund state, blood in stool, difficulty in breathing, severe tremors and fits, and infection were euthanized by carbon dioxide overdosing upon the recommendation of the house veterinarian. The survival of the animals in the control and treated groups was determined daily during the 30-day period of observation and animals were sacrificed on the last day. Animals in the pre-treatment group were sacrificed on day 15.

### Complete blood count and plasma preparation

At the day of sacrifice, blood from each control and treated animal was collected in EDTA-treated tubes by heart puncture. Briefly each animal was anaesthetized with isoflurane in a chamber of 3% isoflurane. The chest fur of the anaesthetized animal was trimmed off and the heart was located by feeling for the heart beat. Cardiac puncture was performed using a 2-ml syringe with a 22-gauge needle. The drawn blood was gently transferred to the EDTA-treated tube. Complete blood count was carried out on blood of Control-1, Control-2, and DAA-I-treated animals using the Abbott Cell-Dyn 3700 Hematology Analyzer. White blood cell, lymphocyte, monocyte and platelet were summarized and compared. Plasma from each mouse was prepared by centrifuging the collected blood at 2,000 x g for 10 minutes in a refrigerated centrifuge. The plasma samples were stored at –20°C until use.

### Assay of IL-6 and TNF- α in plasma

The content of IL-6 and TNF-α in the plasma was assayed using ELISA kitsfrom R&D system, USA. Briefly, 100 μL of plasma or standard solution were added to a 96-well plate, which was coated by the capture antibodies and blocked by 1% BSA. After incubation for 2 hours, the plate was washed and the detection antibody was added. A working solution of streptavidin-HRP, substrate and stop solution, were sequentially added, and the optical density at 450nm was recorded.

### Assay of PGE_2_ in plasma

The level of PGE_2_ and its metabolites in the plasma of each mouse were measured using a Cayman Prostaglandin E Metabolite EIA (Item No. 514531) from Cayman Chemical Company according to the manufacturer’s instructions. The Cayman’s assay is based on the competition between a stable PGE metabolite derivative, PGEM, and a PGEM-acetylcholinesterase (AChE) conjugate (PGEM tracer) for a limited number of PGEM-specific rabbit antiserum binding sites. The PGE_2_ and its metabolites in plasma were first converted to PGEM. Briefly,each 100-μL plasma and standard solution was incubated overnight, at 37°C, with 30 μL of 1M carbonate buffer. Forty μL of 1M phosphate buffer and 30μL of EIA buffer were added to each plasma or standard solution. A 96-well plate was pre-coated with mouse monoclonal anti-rabbit IgG, and incubated with tracer, antiserum, and either treated plasma samples or standard solutions. After an overnight incubation, the plate was washed and 200 μl Ellman’s reagent was added. The optical density at 405nm was recorded using a microplate reader.

### DAA-I and plasma PGE_2_

A separate procedure was developed to study the effect of DAA-I on the plasma level of PGE_2_ in normal mice. Thirty nine mice were separated into two groups. One group was administered, by gavage, 0.1 ml of water and the other 0.1 ml DAA-I solution equivalent to 800 nmole/kg body weight per day for 28 days. The animals were kept for another 14 days for post-DAA-I effect. Three animals were sacrificed on Day 0 to serve as starting control. Three animals from each group were sacrificed on Day 7, 14, 15, 28, 35, and 42. Blood from each animal was obtained by cardiac puncture and plasma was prepared as described above. The level of PGE_2_ in the plasma samples was determined as described above. An addition experiment to study the effect of losartan on the action of DAA-I was also carried out. In this experiment, 3 groups of 3 mice were administered (i) vehicle (ii) 800 nmole/kg DAA-I, and (iii) 800 nmole/kg plus 50 nmole/kg losartan, respectively, for a period of 30 days. Animals were sacrificed on day 30 and their circulating PGE_2_ was determined as described above.

### Statistical analysis

Each value was expressed as a mean ± S.E.M. Comparisons between groups was analyzed by One-Way ANOVA followed by Tukey’s post-hoc test. p < 0.05 was considered as statistically significant.

## Results

**Fig 1:**
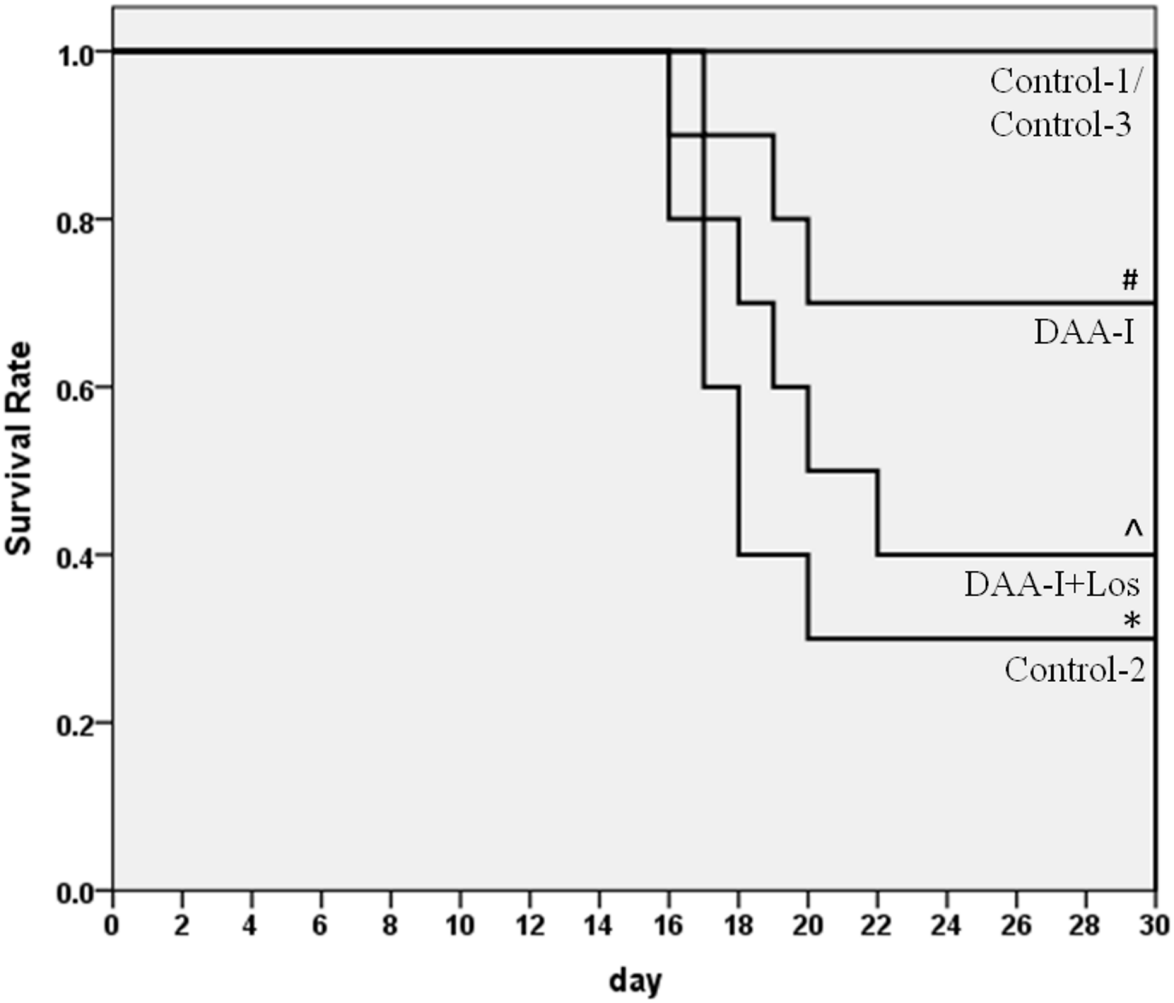
Kaplan-Meier curve of the effect of DAA-I and DAA-I plus losartan on the survival of mice exposed to 6.4Gy gamma irradiation. Animals were administered daily vehicle or drug/s on day 1 for a period of 30 days. Control-1: animals were administered 0.1 ml vehicle (water) and were not irradiated. Control-2: animals were administered vehicle and were irradiated. Control-3: animals were administered 800 nmole/kg DAA-I and were not irradiated. DAA-I: animals were administered 800 nmole/kg DAA-I and were irradiated. DAA-I + Los: animals administered 50 nmole/kg losartan and 800 nmole/kg DAA-I and were irradiated.* p< 0.05 vs Control-1. ^#^ p<0.05 vs Control-2. ^ʌ^ p<0.05 vs Control-3.

**Fig 2:**
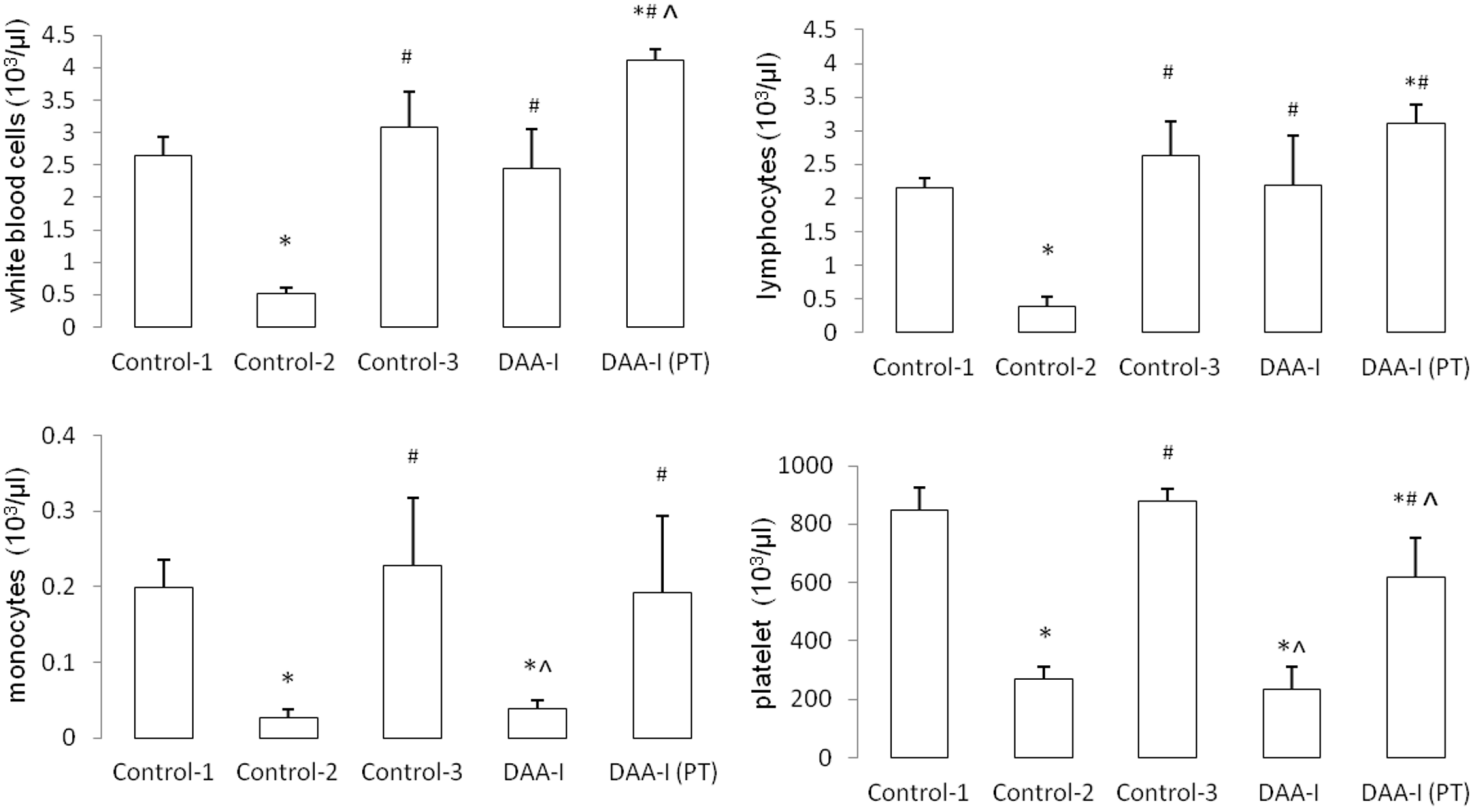
The effects of DAA-I on the profile of leucocytes and platelet in gamma irradiated mice. Control-1: animals were administered 0.1 ml vehicle (water) and were not irradiated. Control-2: animals were administered vehicle and were irradiated. Control-3: animals were administered 800 nmole/kg DAA-I and were not irradiated. DAA-I: animals were administered 800 nmole/kg DAA-I and were irradiated. DAA-I(PT): animals were administered 800 nmole/kg DAA-I for 14 days before TBI and a further 15 days post TBI. Each value is a mean ± S.E.M. obtained from 3 mice. Comparisons between groups was analyzed by One-Way ANOVA followed by Tukey’s post-hoc test. * p< 0.05 vs Control-1. ^#^ p<0.05 vs Control-2. ^ <0.05 vs Control-3.

**Fig 3:**
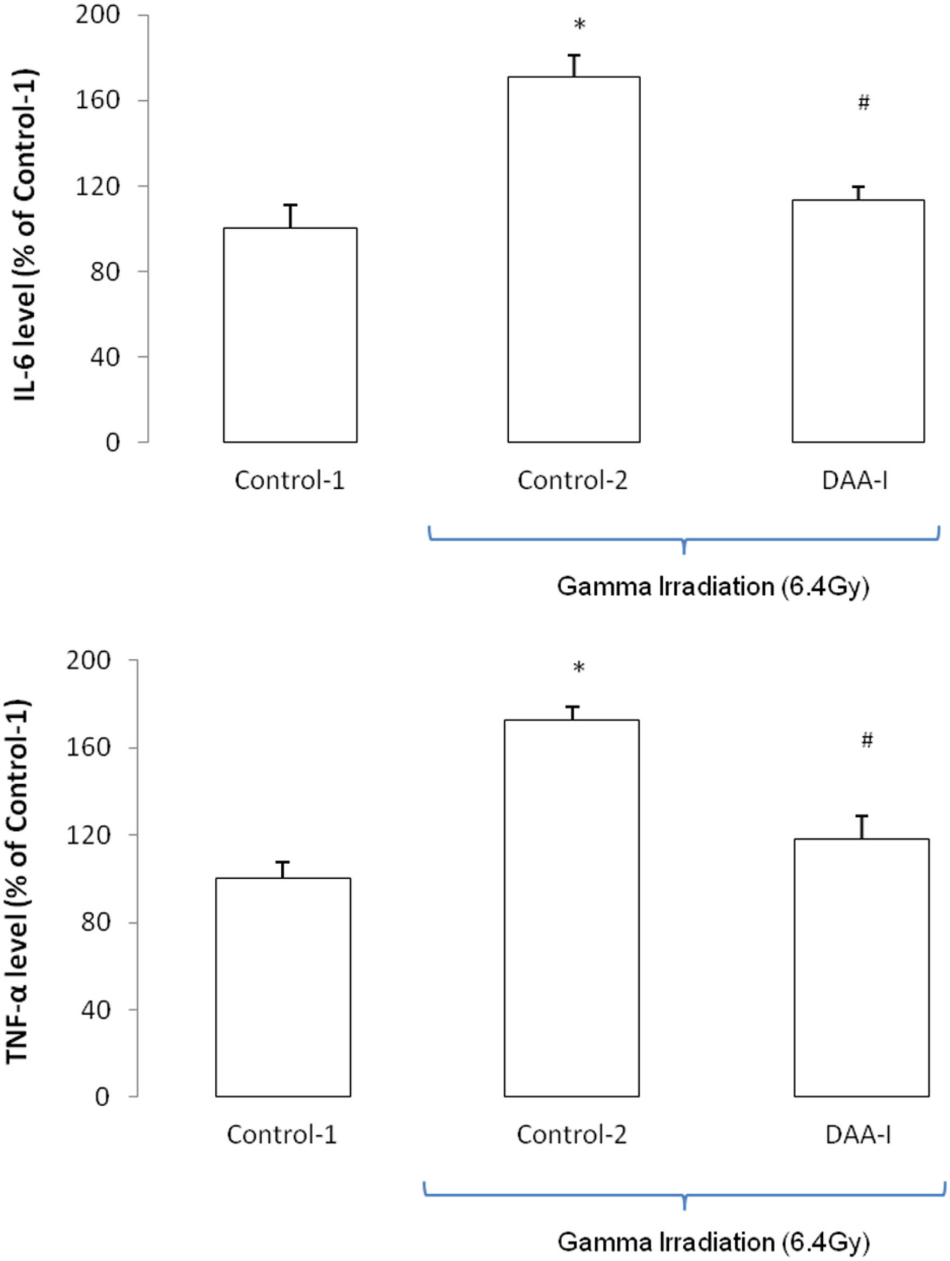
Effects of DAA-I on expression of IL-6 andTNF-α in plasma of gamma irradiated mice.. Control-1: animals were administered 0.1 ml vehicle (water) and were not irradiated. Control-2: animals were administered vehicle and were irradiated. DAA-I: animals were administered 800 nmole/kg DAA-I and were irradiated. Each value was a mean ± S.E.M. obtained from 3 separate animals. Comparison between groups was analyzed by One-Way ANOVA followed by Tukey’s post-hoc test. * p < 0.05 vs Control-1. ^#^ p < 0.05 vs Control-2.

Fig 1 shows that 6.4 Gy of TBI caused a 70% fatality in mice. DAA-I significantly protected the mice from the lethality of TBI, and losartan attenuated the protection. Six animals in the Conrol-2 group were humanely euthanized, and 1 animal died (overnight) without euthanasia. Three animals in the DAA-I-treated group were euthanized. Four animals in the DAA-I and losartan-treated group were euthanized, and 2 animals died (overnight) without euthanasia. All the 6 mice that were pre-treated with DAA-I for 14 days survived the 15-day period of post TBI. Surviving TBI mice that were not treated with DAA-I (Control-2) developed leucopenia, lymphocytopenia, monocytopenia and thrombocytopenia. DAA-I, administered post TBI attenuated the leucopenia and lymphocytopenia but not the monocytopenia and thrombocytopenia (Fig 2). However, in mice that were treated with DAA-I for 14 days before and 15 days after TBI, significant recovery of the four myelodysplastic syndromes were observed (Fig 2).TBI also caused an increase in circulating IL-6 and TNF-α in surviving mice, and DAA-I attenuated the increase of both the inflammatory cytokines (Fig 3).

**Fig 4:**
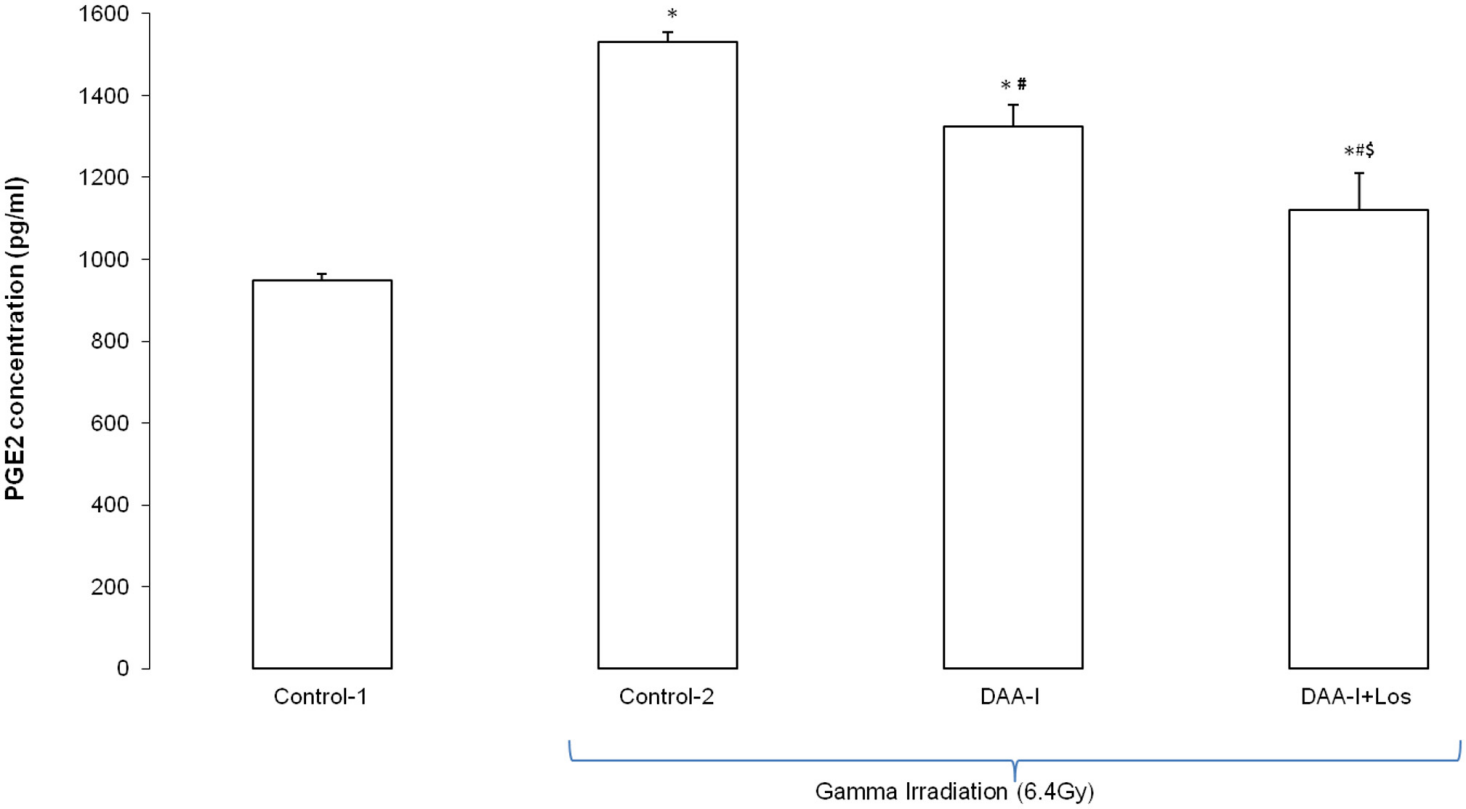
Effect of DAA-I and DAA-I plus losartan on the plasma level of PGE_2_ in gamma irradiates mice. Control-1: animals were orally administered 0.1 ml vehicle (water) and were not irradiated. Control-2: animals were orally administered 0.1 ml vehicle and were irradiated. Each value was a mean ± S.E.M. obtained from 3 separate animals. Comparisons between groups was analyzed by One-Way ANOVA followed by Tukey’s post-hoc test. * p< 0.05 vs Control-1; ^#^ p<0.05 vs Control 2; ^$^ p<0.05 vs DAA-I 6.4Gy.

**Fig 5:**
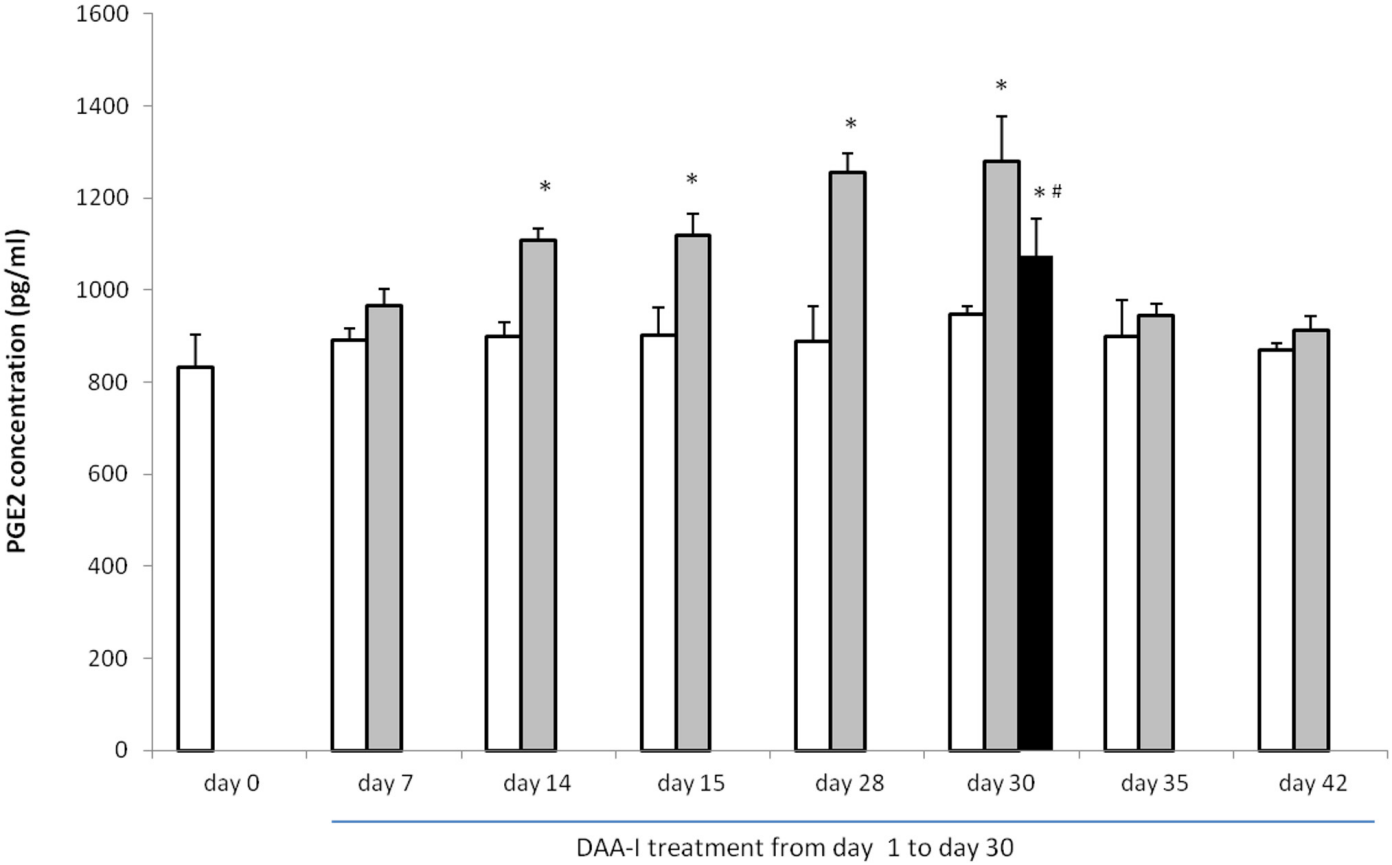
Temporal profile of circulating PGE_2_ in mice treated with 800 nmole/kg DAA-I. Hollow histograms represent values obtained from mice that were administered vehicle, light solid filled histograms represent values obtained from mice that were administered DAA-I, and the dark solid filled histogram represents value obtained from mice that were administered DAA-I and losartan. Three animals were sacrificed at 1 hour post DAA-I or vehicle administration on the day indicated except on day 15 where the animals were sacrificed without DAA-I or vehicle administration. Each value is a mean ± S.E.M. obtained from 3 animals. Comparisons between groups were analyzed by One-Way ANOVA followed by Tukey’s post-hoc test. * p< 0.05 vs day 0; ^#^ p<0.05 vs DAA-I day 30.

Fig 4 shows that the level of circulating PGE_2_ in the surviving gamma irradiated mice was significantly greater than the level in the non irradiated control animals. However, the levels of circulating PGE_2_ varied significantly among the three groups of surviving mice. The level in the three surviving animals that were administered water had the highest level, and this was followed by the level in the 3 randomly selected mice (out of the 7 surviving mice) that were administered DAA-I, and the level in the 3 randomly selected mice (out of 4 surviving mice) that were administered DAA-I and losartan. Chronic administration of 800 nmole/kg/day for 30 days caused a time-dependent increase in circulating PGE_2_ in mice (Fig 5). Significant increase was observed on day 14 to day 30. Animals in this experiment were sacrificed 1 hour after the daily DAA-I administration, except on day 15 where the animals were sacrificed without administration of the 15^th^ day dose. This was carried out to show that the increase in circulating PGE_2_ was not an immediate response to DAA-I administration but was a result of chronic DAA-I administration. Level of circulating DAA-I reverted to basal value a week after stoppage of DAA-I administration indicating that the effect of DAA-I was reversible. Losartan at 50 nmole/kg significantly attenuated the increase in circulating PGE_2_ caused by DAA-I showing that the action of DAA-I was mediated via the angiotensin AT_1_ receptor.

## Discussion

Attenuation of basal production of angiotensin II by ACE inhibitors or blockade of the action of locally produced angiotensin II by ARBs was found to be effective in preventing the development and/or mitigating the severity of radiation-induced injury in the kidney, lung and brain [14–16]. This link between a reduced functional angiotensin II and a reduction in radiation-induced late injuries is also seen in the present radioprotection findings on DAA-I. At a dose of 800 nmole/kg, DAA-I significantly increased the survival of mice exposed to a lethal dose of TBI. In common with ACE inhibitors and ARBs, DAA-I attenuates the action of angiotensin II. It does so by acting as an agonist on the angiotensin AT_1_ receptor and mediates responses opposing the deleterious effects of angiotensin II [6–13]. As a result the radioprotective action of DAA-I was blocked by a concurrently administered dose of 50 nmole/kg losartan. Losartan at this dose had been shown, in an early study, to be sub-pharmacological [7]. Furthermore, the radioprotection correlated to an increase in PGE_2_ in the plasma of surviving animals, and losartan-treated surviving animals had the lowest level of plasma PGE_2_. At this junction, it is interesting to note that the 3 surviving non DAA-I treated gamma irradiated-mice had the highest level of plasma PGE_2_. Although the exact cause for this observation is not known, it could be a reflection of the different processes that govern the level of plasma PGE_2_ in the DAA-I-treated and non treated groups. It could also be attributed to the fact that the 3 surviving animals were the natural survivors (out of the 10 mice in the group) that were able to produce high plasma PGE_2_ for radioprotection; and even if similar number of natural survivors were present in the DAA-I-treated group the average plasma level of PGE_2_ in this group would still be lower than the non DAA-I-treated group as the DAA-I-treated group consisted of 4 additional mice that were not natural survivors but survived because of DAA-I treatment. The latter is a more plausible explanation as the average value of the 4 surviving mice in the DAA-I plus losartan group was significantly the lowest of the three groups. This observation could also indicate that plasma PGE_2_ is a survival biomarker, and the ability to generate a minimum level with or without drug intervention protects the animal from irradiation.

The direct effect of DAA-I on the profile of plasma PGE_2_ was separately studied using normal mice, where the animals were treated with the same dose (800 nmole/kg) of DAAI for up to a period of 30 days. The response to DAA-I was temporal and significant increase in plasma PGE_2_ was observed on day-14 of DAA-I administration. Concurrent administration of losartan for 30 days, significantly reduced DAA-I-induced increase in plasma PGE_2_ confirming that DAA-I mediates plasma PGE_2_ increase via the angiotensin AT_1_ receptor. The effect waned off within a week after cessation of DAA-I treatment. These findings are in agreement with those of earlier studies showing that the actions of DAA-I were losartan- and indomethacin-sensitive [7, 8, 11, 13, 19, 20].

PGE_2_ acts on its receptor, which exists as four subtypes (EP1 to EP4) and produces a variety of responses that blur the line between it being categorized as a pro-inflammatory or anti-inflammatory molecule [21]. The recent discovery of two isoforms of PGES (PGE_2_ synthase) [22, 23], and a finding that DAA-I selectively induced a COX-1 dependent production of PGE_2_ in HUVEC that was inhibited by losartan [6] may provide an explanation for our suggestion that PGE_2_ mediates the radioprotective action of DAA-I seen in the present study. Of the two isoforms of PGES, mPGES1 (membrane PGE_2_ synthase 1) is an inducible enzyme distinctly coupled to COX-2, and has been shown to be markedly induced by proinflammatory stimuli that trigger the production of PGE_2_ that contributes to inflammation [24, 25]. cPGES (cytosolic PGE_2_ synthase) is an ubiquitous constitutively expressed enzyme that is stringently coupled to constitutively expressed COX-1. It is known to contribute physiologically to the production of PGE_2_ required for the maintenance of tissue homeostasis [24, 25]. These findings suggest that PGE_2_ formed from the action of DAA-I on the angiotensin AT_1_ receptor was likely to be via the COX-1-cPGES pathway. The PGE_2_ formed was not involved in the mediation of inflammation caused by the gamma radiation but mediated the radioprotection action of DAA-I. This suggestion is supported by the finding of Cohen et al. showing that PGE_2_ produced through COX-1 protected jejunum crypt stem cell survival and proliferation in gamma irradiated mice [18], and that of Houchen et al., showing that COX-1^−1-^ but not COX-2^−1-^ mice has increased crypt epithelial cell apoptosis and decreased clonogenic stem cell survival after gamma irradiation [26]. The effects of PGE_2_ on both crypt epithelial apoptosis and intestinal crypt stem cell survival are mediated through the EP_2_ receptor, which is expressed primarily in the nuclei [27].

The circulating level of two inflammatory cytokines, TNFα and IL-6, was elevated in surviving gamma irradiated mice, and DAA-I treatment attenuated the level. PGE_2_ has been shown to either inhibit expression or the action of a number of inflammatory cytokines [28–30], and the PGE_2_ released by DAA-I treatment was likely responsible for the attenuation of the two inflammatory cytokines. The antioxidants, ferulic acid and eipcatechin, that attenuated the inflammation of gamma irradiated-mice had also been shown to reduce the circulating level of these two cytokines [1, 4].

Hematopoietic syndrome is a major cause of death in total-body irradiated mice, and occurs within 30 days after exposure [31–33]. In the present study, the profile of white blood cell, lymphocyte, monocyteand platelet in surviving gamma irradiated-mice was investigated 30 days after TBI. Surviving mice were found to suffer from leucopenia, lymphocytopenia, monocytopenia and thrombocytopenia. Treatment with DAA-I during the 30 days post TBI attenuated the leucopenia and lymphocytopenia but not the monocytopenia and thrombocytopenia. However, when animals were pre-treated with DAA-I for a period of 14 days before TBI and a further 15-days post TBI, significant recovery in the number of the total white blood cell, lymphocyte, monocyte and platelet was observed in the surviving animals. The 14-day pre-treatment period corresponded to the period that resulted in a significant rise in circulating PGE_2_ in DAA-I-treated mice, and this strongly suggests that PGE_2_ could played a major role in the recovery of each white blood cell type. This assumption is supported by recent findings showing that a long-acting PGE_2_ analogue (dmPGE_2_) administered intraperitoneally immediately after TBI decreased the loss of functional hematopoietic stem and progenitor cells that resulted from the radiation [34]. In an *in vivo* situation, the PGE_2_ that was formed as a result of 14-day DAA-I pre-treatment would be equivalent to the PGE_2_ that was immediately administered after TBI, and both would equally protect hematopoietic cells from gamma radiation. Bone marrow cells released PGE_2_ as an immediate response to gamma irradiation, and the released PGE_2_ triggered its own production in surviving cells, which would further aid hematopoietic recovery [33]. Hence exogenous PGE_2_ and PGE_2_ formed from DAA-I pre-treatment would also enhance and potentiate the innate response of locally produced PGE_2_. This could be a contributing factor in the observation that pre-treatment followed by post-treatment of DAA-I accorded wider protection against bone marrow injury than just post-treatment in the gamma irradiated mice.

ACE inhibitors and ARBs have been shown to reduce hematopoietic toxicity and improve survival in mice exposed to a lethal dose of gamma radiation. These two classes of drug reduced the activity of local angiotensin II, and a reduction of the proliferative action of angiotensin II on hematopoietic cells that triggers the cells to enter the cell cycle and become susceptible to radiation injury was suggested as the mechanism of action of the ACE inhibitor and ARBs investigated [35, 36]. ACE inhibitors and ARBs had also been shown to be effective radioprotectors in other organ systems, which include the brain, kidney, and lung [37, 32]. These two drugs were shown to curtail the actions of angiotensin II-linked inflammatory pathways that were supposed to be upregulated by gamma radiation. Thus the restoration of (i) cognitive impairment of gamma irradiated rat was related to the restoration of the level of Homer 1a in the hippocampus and cortex of the brain [16], (ii) radiation-induced pneumopathy and pulmonary fibrosis corresponded to the restoration of increased TGF ß-1 mRNA and over expression of α SMA in the lung [15], (iii) renal glomerular and tubule-interstitial function by suppression of the renin-angiotensin system despite the fact that no evidence exists to date of the activation of the renin-angiotensin system by irradiation [38]. Despite these findings, the specific causal relationship between angiotensin II activity and radiation injury remains unknown, and systemic administration of angiotensin II has even been shown to accelerate hematopoietic recovery after irradiation [39]. Hence, the exact involvement of angiotensin II in radiation injury and the radioprotective efficacy of ACE inhibitors and ARBs need further investigation. The specific actions of DAA-I, described in the present study, provides a novel alternative measure of radioprotection that bypasses the involvement of angiotensin II. In addition, DAA-I is active orally and possesses anti-oxidant and anti-inflammatory actions, which could add to its efficacy as a novel radioprotector.
